# French Veterinary Congress of 1900

**Published:** 1900-07

**Authors:** 


### FRENCH VETERINARY CONGRESS OF 1900.

A large number of subscriptions from individuals and societies have been received, and the success of the Congress is assured. All expecting to attend the Congress are requested to send notice as soon as possible, at least one month in advance of the meeting. This information is desired in order that the committee may know how many preliminary programmes to send out, and that they may give to the railroads a complete list with the demand for reduced rates in fare. The State has given 2000 francs for the meeting, and different societies have subscribed 1305 francs. Some 340 French veterinarians have joined, and 46 foreign up to June 20th. The foreign delegates are from Germany, Belgium, Cuba, Egypt, Denmark, Spain, United States, Hungary, Italy, Luxemburg, Portugal, Argentine Republic, Switzerland, and Uraguay. The United States has only one delegate, Dr. A. Liautard, of New York.

ARMY legislation will be a topic of earnest consideration at Detroit. The failure of our bill in the House at Washington will be a source of discussion, and led by that foremost advocate of army recognition of rank, Dr. Rush S. Huidekoper, it is sure to lead up to the final achievement of this much-to-be-desired end. The next Congress will determine beyond peradventure this long-considered question, and how we can work most effectively in securing favorable consideration will be the topic presented by that able leader in this work, through whose directing efforts our bill passed the last Senate in a battle on the floor of that august body.

---

ON another page in this issue will be found a news note of the work of State Veterinarian Pearson in Pennsylvania in again securing for veterinary sanitary control work for the ensuing year some forty thousand dollars. This alone speaks volumes for the success attained in the Keystone State along these lines of work. While almost every other department of the State has been the target of attack by the public press, and gross charges of incompetency, dishonesty, and fraud been openly made, the work of this branch of the Department of Agriculture has gone steadily along with almost the united support of the press, the granges, the breeders' organizations, and the people at large—a just and well-deserved tribute to the efficient work done by the Live-stock Sanitary Board under the wise and conservative direction of Dr. Pearson.

In this work Dr. Pearson has had the assistance of more than one hundred veterinarians throughout the State, and so well has the work of this large number been directed and regulated that only the most commendable expressions of satisfaction have been heard from every section of our State. What has been done well in Pennsylvania can be equally well accomplished in other States.

---

THE Pennsylvania State Live-stock Sanitary Board held a meeting at Harrisburg, in the office of the Governor, on July 6th. John Hamilton, Secretary of Agriculture, was elected treasurer of the Board, and Jesse K. Cope, Dairy and Food Commissioner, was elected Vice-President. Governor Stone and State Veterinarian Pearson continue as President and Secretary, respectively. The work of the year terminating May 31st was gone over in detail, and it was shown that tuberculosis is being repressed at a very encouraging rate, and that the live-stock owners throughout the State are working in thorough sympathy and accord with the Board.

It was arranged that hereafter cattle brought into the State and found upon test to be tuberculous should be returned to the State whence they came only when special authority for such return shipment is granted by the State Veterinarian.

It was arranged to devote $40,000 to field work—to the practical work of suppressing disease—during the year beginning June 1, 1900, and terminating May 31, 1901.

---

Dr. Mazyck P. Ravenel, Bacteriologist of the State Livestock Sanitary Board of Pennsylvania, and D. J. McCarthy, M.D., would be glad to have material sent them for examination, and want especially diseases simulating hydrophobia, and nervous diseases of all kinds, in order to prove whether or not these changes are found only in rabies. They request that the head shall be cut off about the middle of the neck and packed in ice, or else put in 10 per cent. formalin solution, and forwarded to Dr. Leonard Pearson, 3608 Pine Street, Philadelphia. They request that in no case should the suspected animal be destroyed by chloroform, owing to the production of changes in the nervous system which would confuse the findings of such an examination.

